# *Legionella dumoffii* Utilizes Exogenous Choline for Phosphatidylcholine Synthesis

**DOI:** 10.3390/ijms15058256

**Published:** 2014-05-09

**Authors:** Marta Palusinska-Szysz, Agnieszka Szuster-Ciesielska, Magdalena Kania, Monika Janczarek, Elżbieta Chmiel, Witold Danikiewicz

**Affiliations:** 1 Department of Genetics and Microbiology, Institute of Microbiology and Biotechnology, Maria Curie-Sklodowska University, Akademicka 19 St., 20-033 Lublin, Poland; E-Mails: mon.jan@poczta.umcs.lublin.pl (M.J.); elawisniewska87@gmail.com (E.C.); 2 Department of Virology and Immunology, Institute of Microbiology and Biotechnology, Maria Curie-Sklodowska University, Akademicka 19 St., 20-033 Lublin, Poland; E-Mail: aszusterciesielska@gmail.com; 3 Mass Spectrometry Group, Institute of Organic Chemistry Polish Academy of Sciences, Kasprzaka 44/52 St., 01-224 Warsaw, Poland; E-Mails: magdalena.kania@icho.edu.pl (M.K.); witold.danikiewicz@icho.edu.pl (W.D.)

**Keywords:** LC/ESI-MS, MALDI-TOF, *Legionella dumoffii*, phosphatidylcholine, TNF-α

## Abstract

Phosphatidycholine (PC) is the major membrane-forming phospholipid in eukaryotes but it has been found in only a limited number of prokaryotes. Bacteria synthesize PC via the phospholipid *N*-methylation pathway (Pmt) or via the phosphatidylcholine synthase pathway (Pcs) or both. Here, we demonstrated that *Legionella dumoffii* has the ability to utilize exogenous choline for phosphatidylcholine (PC) synthesis when bacteria grow in the presence of choline. The Pcs seems to be a primary pathway for synthesis of this phospholipid in *L. dumoffii*. Structurally different PC species were distributed in the outer and inner membranes. As shown by the LC/ESI-MS analyses, PC15:0/15:0, PC16:0/15:0, and PC17:0/17:1 were identified in the outer membrane and PC14:0/16:0, PC16:0/17:1, and PC20:0/15:0 in the inner membrane. *L. dumoffii pcsA* gene encoding phosphatidylcholine synthase revealed the highest sequence identity to *pcsA* of *L. bozemanae* (82%) and *L. longbeachae* (81%) and lower identity to *pcsA* of *L. drancourtii* (78%) and *L. pneumophila* (71%). The level of TNF-α in THP1-differentiated cells induced by live and temperature-killed *L. dumoffii* cultured on a medium supplemented with choline was assessed. Live *L. dumoffii* bacteria cultured on the choline-supplemented medium induced TNF-α three-fold less efficiently than cells grown on the non-supplemented medium. There is an evident effect of PC modification, which impairs the macrophage inflammatory response.

## 1. Introduction

*Legionella* are Gram-negative bacilli that are highly successful in colonizing natural and artificial aquatic environments. Dissemination in the environment is facilitated by their characteristic biphasic lifestyle; *Legionella* may adapt to distinct intracellular and aquatic environments by alternating between a “replicative” and a “virulent” form in response to growth conditions. In water systems, *Legionella* infects and replicates within protozoa, colonize surfaces, and grow in biofilms [1]. Bacteria enclosed in water-air aerosol are inhaled into the lower respiratory tract and subsequently engulfed by enteric pulmonary macrophages. The capability of *Legionella* of intracellular proliferation in immune cells designed to kill bacteria and using them as their host cell is crucial for development of pneumonia known as Legionnaires’ disease. Currently, the family *Legionellaceae* is composed of 58 species isolated from environmental sources, but 21 of them have been isolated from humans [2,3]. Among *Legionella* species that cause human pneumonia, *L. pneumophila* is the most common causative agent, while *L. dumoffii* is the fourth [4]. Pneumonia caused by *L. dumoffii* is rapidly progressive and fulminant owing to the ability of this bacterium to invade and proliferate in human alveolar epithelial cells [5]. The disease is often fatal, especially in immunocompromised patients. *L. dumoffii* has been isolated from pericarditis, prosthetic valve endocarditis, and septic arthritis, which indicates that *L. dumoffii* is responsible for extrapulmonary infections [6,7].

The way of bacterial penetration into the host cell and factors indispensable for settling the specific microniche, *i.e.*, the digestive vacuole, are dependent on virulence factors released from the cell, unique properties of surface components, and the ability to utilize host metabolites. Crucially for the biogenesis and maintenance of the bacterial replicative vacuole, *L. pneumophila* uses a type IV secretion system (Dot/Icm) to deliver a large number of effector proteins to the host cell [8]. The coordinate actions of the bacterial effectors allow *Legionella* to subvert innate immune response and evade host destruction. Moreover, the components of the cell envelope: proteins, peptidoglycan, lipopolysaccharide (LPS), and phospholipids participate in the highly specific *Legionella*-host interactions. *Legionella* phospholipids are characterized by a high phosphatidylcholine (PC) content, which is untypical of bacteria and specific for a narrow group of pathogenic and symbiotic microbes whose life cycle is strictly associated with eukaryotic cells. PC is a major component of eukaryotic cell membranes and plays a significant role in signal transduction. The high PC content in intracellular membranes of pathogens, such as *Legionella*, makes the cells of the microbes similar to the host cells. Bacteria synthesize PC via two different routes, *i.e.*, the phospholipid *N*-methylation (Pmt) or the phosphatidylcholine synthase (Pcs) pathway. In the Pmt pathway, phosphatidylethanolamine is methylated three times to yield PC in reactions catalyzed by one or several phospholipid *N*-methyltransferases (PMTs). In the Pcs pathway, choline is condensed directly with CDP-diacylglyceride to form PC in a reaction catalyzed by a bacterium-specific Pcs enzyme [9]. Since choline is not a biosynthetic product of prokaryotes, the Pcs pathway is probably a direct sensor of environmental conditions, using choline availability as an indicator of the status of the location in which the bacterium is found. It has been shown that *L. pneumophila* and *L. bozemanae* are capable of utilisation of exogenous choline for PC synthesis [10,11]. Apart from the structural role, the exact function of PC in *Legionella* cells remains unexplained. However, PC-deficient mutants of *L. pneumophila* exhibited attenuated virulence and increased susceptibility to macrophage-mediated killing. These defects were attributed to reduced bacterial binding to macrophages and a poorly functioning Dot/Icm system. In the process of binding to macrophages, *L. pneumophila* uses the platelet-activating factor receptor (PAF receptor), which harbors the same glycerophosphocholine head group as PC. Due to this structural similarity, PC is required for efficient binding of *L. pneumophila* to macrophages via the PAF receptor [12].

In response to infection caused by *Legionella*, macrophages produce inflammatory cytokines, such as interleukin 6 (IL-6), interleukin 1α (IL-1α), interleukin 1β (IL-1β), interleukin 12 (IL-12), interferon γ (INF γ), and tumor necrosis factor α (TNF-α) [13]. Among these cytokines, TNF-α appears to be pivotal for activation of phagocytes and resolution of pneumonic infection [14,15]. Treatment of rat alveolar macrophages with TNF-α resulted in decreased intracellular growth of *L. pneumophila* [16]. In turn, inhibition of endogenous TNF-α activity via TNF-α-neutralizing antibodies resulted in enhanced growth of *L. pneumophila* in the mouse lung [17]. However, little is known about how the pathogen PC influences the induction of proinflammatory cytokines in the host.

The aim of our study was to investigate the ability of *L. dumoffii* to utilize exogenous choline for PC synthesis using Matrix-Assisted Laser-Desorption/Ionization (MALDI)-Time of Flight (TOF) MALDI/TOF and Liquid Chromatography Coupled with the Mass Spectrometry Technique Using the Electrospray Ionization Technique (LC/ESI-MS) techniques. We also wanted to determine whether the bacteria use the Pcs pathway for PC synthesis by identification of a *pcsA* gene encoding phosphatidylcholine synthase. Next, the correlations between the PC species content in the *L. dumoffii* membranes and the level of TNF-α produced by human macrophages were investigated.

## 2. Results

### 2.1. Utilization of Exogenous Choline by L. dumoffii

**Figure 1.**
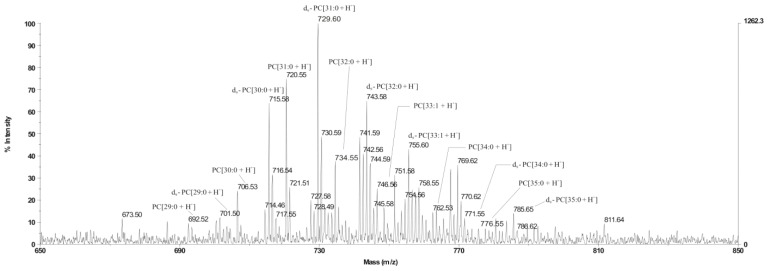
Positive ion mode MALDI-TOF spectrum of the PCs of *L. dumoffii* cultured on the medium with labeled choline: *m*/*z* = 692.52*–*785.65. All peaks were marked according to their *m*/*z* ratio.

In order to study the ability of *L. dumoffii* to use exogenous choline for PC synthesis, the bacteria were grown on BCYE medium supplemented with deuterium-labeled choline. Lipids isolated from the cells were analyzed using MALDI-TOF mass spectrometry with a CHCA matrix (in the reflectron mode). The positive ionization MALDI-TOF spectrum contained a cluster of molecular ions at *m*/*z*: 692.52–785.65 corresponding to PC species (Figure 1). Within the cluster, protonated ions were identified at *m*/*z* 692.52 PC[29:0 + H]^+^, 706.53 PC[30:0 + H]^+^, 720.55 PC[31:0 + H]^+^, 734.55 PC[32:0 + H]^+^, 746.56 PC[33:1 + H]^+^, 762.53 PC[34:0 + H]^+^, 776.55 PC[35:0 + H]^+^, and corresponding ions with mass higher by 9 at *m*/*z* 701.50 d_9_-PC[29:0 + H]^+^, 715.58 d_9_-PC[30:0 + H]^+^, 729.60 d_9_-PC[31:0 + H]^+^, 743.58 d_9_-PC[32:0 + H]^+^, 755.60 d_9_-PC[33:1 + H]^+^, 771.55 d_9_-PC[34:0 + H]^+^, 785.65 d_9_-PC[35:0 + H]^+^. Deuterium-labeled PC[31:0 + H]^+^ was observed as the dominant constituent of the extract. The presence of labeled PCs in the MALDI-TOF spectrum showed that *L. dumoffii* was able to utilize exogenous choline for PC synthesis.

### 2.2. Membrane Localization of PC

#### 2.2.1. Membrane Fractionation

**Figure 2.**
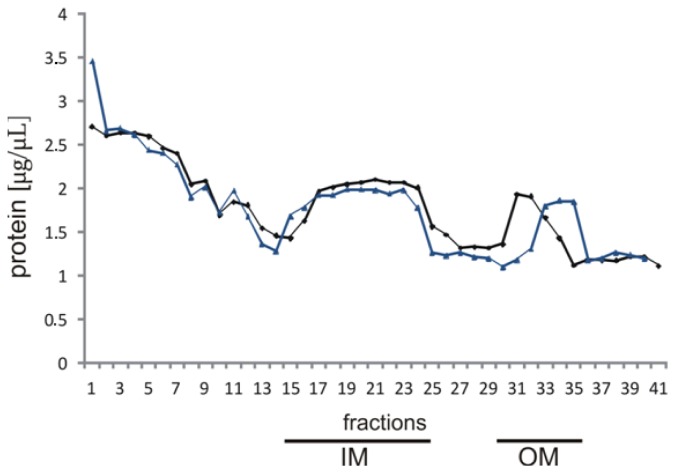
Separation of *L. dumoffii* inner (IM) and outer membrane (OM) by discontinuous sucrose density gradient centrifugation. Fractions of 1 mL were collected from the top of the gradient and assayed for the presence of protein (μg/μL). Black line—for bacteria cultured on medium non-supplemented with choline; Blue line—for bacteria cultured on medium supplemented with choline.

To determine PC localization in the *L. dumoffii* membranes, separation of outer and inner membranes from bacteria cultured with and without labeled choline was performed by sucrose density gradient ultracentrifugation. The protein concentration in the individual fractions showed two major peaks with maxima in fractions 20–22, 31, and 32 for the choline non-supplemented bacteria. In the case of choline-supplemented bacteria, the maxima were detected in fractions 19–23 and 33–35 (Figure 2).

To assign the peaks to a membrane compartment, NADH oxidase and esterase activities (as characteristic for the inner and outer membrane, respectively) were determined. NADH oxidase activity was concentrated in pooled fractions corresponding to the inner membrane (fractions 16–24 for choline non-supplemented bacteria and 15–24 for bacteria cultured with choline). NADH oxidase activity was 228 μmol min^−1^·mL^−1^. Esterase activity was concentrated in pooled fractions represented by the outer membrane (30–34 for bacteria cultured without choline and 32–35 for choline-supplemented bacteria). The NADH oxidase and esterase activities detected confirmed the efficiency of fractionation.

#### 2.2.2. Localization of PC in the Outer and Inner Membranes by LC/ESI-MS Analysis

**Figure 3.**
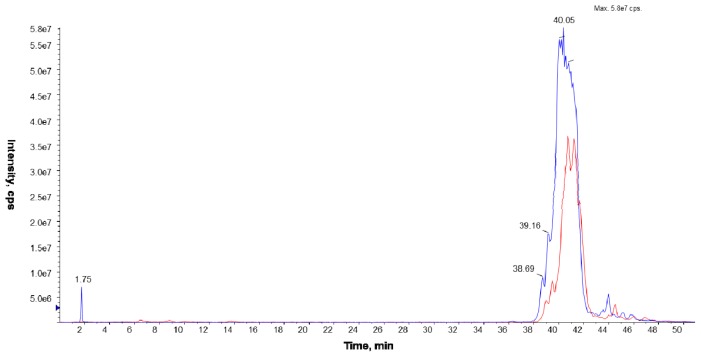
Chromatogram MS obtained with the Precursor Ion Mode for unlabeled (blue; *m*/*z* 184) and labeled (red; *m*/*z* 193) PC molecular species, recorded under NP LC/MS conditions (phase A—hexane/isopropanol (3:2, *v*/*v*) and phase B—an isopropanol/hexane/5 mM aqua solution of ammonium acetate (38:56:5, *v*/*v*/*v*). The following elution program was employed: from 53% B to 80% B for 23 min, 80% B maintained for 4 min, 80% B to 100% B for 9 min, and 100% B maintained for 14 min. The flow rate was 1 mL/min) in the positive ion MS mode; the lipid extract was isolated from the inner cell membrane of *L. dumoffii* cultured on labeled choline.

In the *L. dumoffii* membranes (inner and outer), unlabeled and labeled PC species were identified by the LC/ESI-MS technique using the precursor ion mode (PI) and neutral loss scan (NL) in the positive ion mode. The characteristic fragment of PC compounds at *m/z* 184 corresponding to the polar head group (C_5_H_15_NPO_4_^+^) was employed to determine unlabeled PC using the PI mode, while d_9_-PC compounds were identified by a diagnostic ion of the head group at *m/z* 193 (Figure 3).

In the MS spectra of the analyzed PC compounds, [M + H]^+^ and [M + Na]^+^ ions were observed in the positive ion mode while in the negative ion mode—peaks corresponding to [M + CH_3_COO]^−^ adducts.

**Figure 4.**
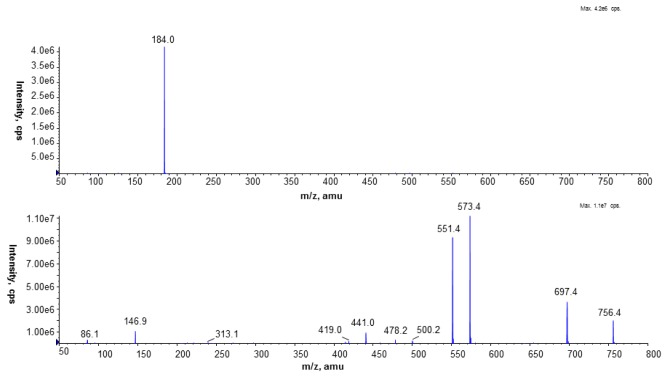
CID spectrum of the PC standard (1,2-dipalmitoyl-sn-glycero-3-phosphocholine) of protonated (top) and sodiated (below) molecules, recorded under NP LC/MS conditions (phase A—hexane/isopropanol (3:2, *v*/*v*) and phase B—an isopropanol/hexane/5 mM aqua solution of ammonium acetate (38:56:5, *v*/*v*/*v*). The following elution program was employed: from 53% B to 80% B for 23 min, 80% B maintained for 4 min, 80% B to 100% B for 9 min and 100% B maintained for 14 min. The flow rate was 1 mL/min) in the positive ion MS mode.

Fragmentation of the PC standard (1,2-dipalmitoyl-sn-glycero-3-phosphocholine; Figure 4) showed that the fragmentation of the sodiated PC molecule gave more information about the structure of the investigated compound. In the CID spectrum of the [M + Na]^+^ ion of the PC standard peaks at *m*/*z* 441.0, 478.2, and 500.2 reflected the presence of palmitic acid in the PC molecule.

Next, neutral loss scanning was applied as an alternative method for the precursor ion mode to identify sodiated PC species in the mixture of lipids isolated from *L. dumoffii* membranes. PC compounds were found in the positive ion mode by the loss of the polar head group with a molecular weight at 183 Da (unlabeled) and 192 Da (d_9_-labeled). According to Hsu and Turk, this phenomenon occurs in two steps through elimination of trimethylamine (59 Da), leading to the loss of C_5_H_14_NPO_4_ (183 Da) from the PC structure [18]. Using the Precursor Ion and Neutral Loss scanning method, numerous PC species were found in the lipid extract obtained from *L. dumoffii* cell membranes. The PI mode seems to be more sensitive than the NL scanning technique for both unlabeled and labeled PC species. On the other hand, the NL method facilitated identification of sodiated PC molecular species in the mixture, which gave more informative CID spectra in the positive ion mode in the fragmentation process.

**Figure 5.**
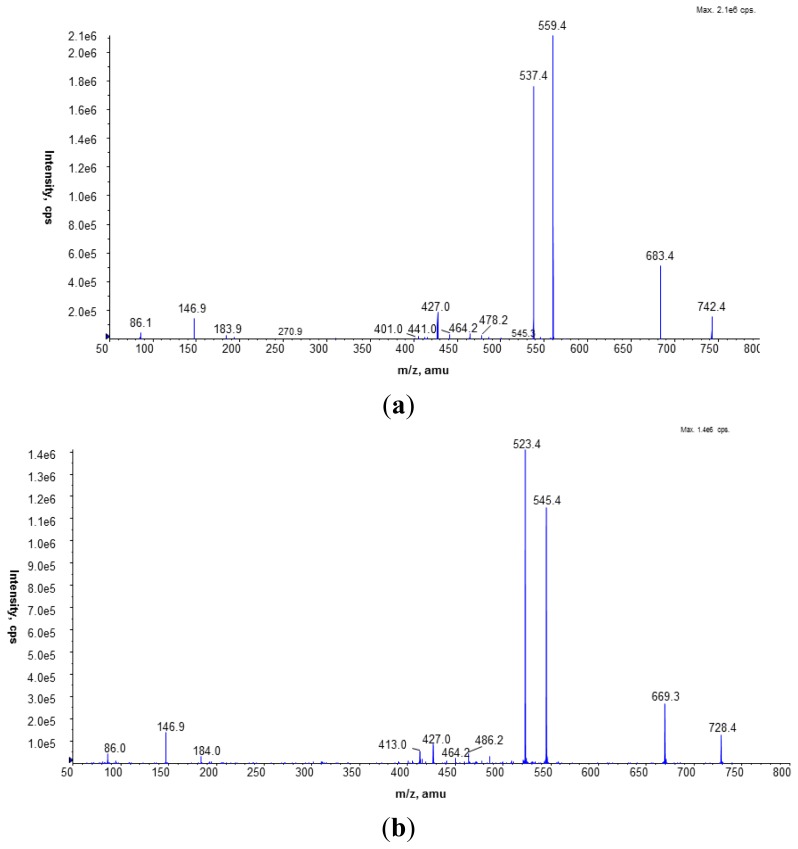
(**a**) CID spectrum of unlabeled 16:0/15:0 PC molecular species identified in the lipid mixture isolated from the inner membrane (*L. dumoffii* cultivated on the medium without labeled choline), recorded under NP LC/MS conditions (phase A—hexane/isopropanol (3:2, *v*/*v*) and phase B—an isopropanol/hexane/5 mM aqua solution of ammonium acetate (38:56:5, *v*/*v*/*v*). The following elution program was employed: from 53% B to 80% B for 23 min, 80% B maintained for 4 min, 80% B to 100% B for 9 min and 100% B maintained for 14 min. The flow rate was 1 mL/min) in the positive ion MS mode; (**b)** CID spectrum of unlabeled PC molecular species with FA combination: 14:0/16:0 and 15:0/15:0 identified in the lipid mixture isolated from the outer membrane (*L. dumoffii* cultivated on the medium without labeled choline), recorded under NP LC/MS conditions (phase A—hexane/isopropanol (3:2, *v*/*v*) and phase B—an isopropanol/hexane/5 mM aqua solution of ammonium acetate (38:56:5, *v*/*v*/*v*). The following elution program was employed: from 53% B to 80% B for 23 min, 80% B maintained for 4 min, 80% B to 100% B for 9 min and 100% B maintained for 14 min. The flow rate was 1 mL/min) in the positive ion MS mode.

The presence of unlabeled and labeled PC molecules in *L. dumoffii* cell membranes was also confirmed by the CID spectra of sodiated PC ions obtained in the positive ion mode. For bacteria cultured on the medium without labeled choline, the major PCs with the following composition of fatty acids: 16:0/15:0, 17:0/15:0, and 16:0/17:1 (or cyclic 17:0, indistinguishable under the present mass spectrometry conditions) were determined in the inner membrane on the basis of the fragmentation spectra of peaks at *m*/*z* 742.4, 756.4, and 768.4 (Figure 5a). In the outer membrane, the major species of PC were diacyl 15:0/15:0, 14:0/16:0 (*m*/*z* 728.4), 16:0/17:1 (or cyclic 17:0) (*m*/*z* 768.8), and 17:0/17:1 (or cyclic 17:0) (*m*/*z* 782.8) (Figure 5b). In the CID spectrum of 16:0/15:0 PC shown in Figure 5a, loss of trimethylamine (59 Da) was observed. The peaks at *m*/*z* 559.4 and 537.4 correspond to the elimination of the PC head group giving ions [M + Na − 183]^+^ and [M + Na − 205]^+^, respectively. The fragment ions reflecting the presence of pentadecanoic (15:0) and palmitic (16:0) acids in the PC structure were found at *m*/*z* 405.2, 427.2, 464.2, 478.2, and 500.3. The fatty acids were eliminated as neutral molecules (*m*/*z* 427.2) or as sodium salts (*m*/*z* 405.2, 464.2, 478.2) from the [M + Na]^+^ and [M + Na − N(CH_3_)_3_]^+^ ions in the fragmentation pathway of PC molecular species in the positive ion mode.

In the case of bacteria cultured on the medium with labeled choline, d_9_-labeled PC molecular species were identified in the inner and outer membranes and their structures were established as shown in Table 1.

**Figure 6.**
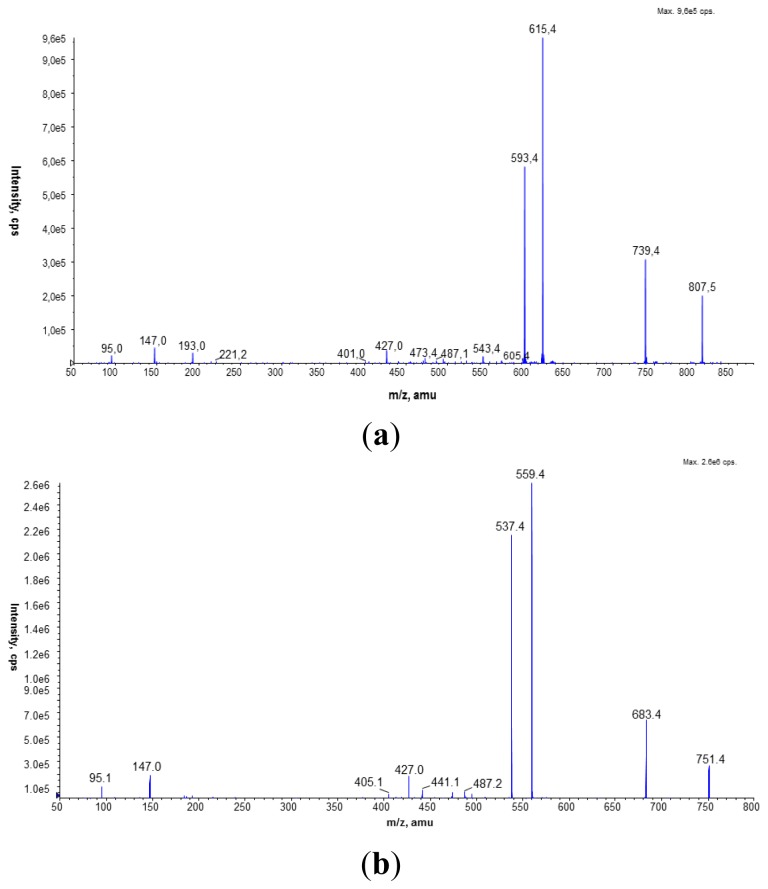
(**a**) CID spectrum of labeled 20:0/15:0 PC molecular species identified in the lipid mixture isolated from the inner membrane (*L. dumoffii* cultivated on the medium with labeled choline), recorded under NP LC/MS conditions (phase A—hexane/isopropanol (3:2, *v*/*v*) and phase B—an isopropanol/hexane/5 mM aqua solution of ammonium acetate (38:56:5, *v*/*v*/*v*). The following elution program was employed: from 53% B to 80% B for 23 min, 80% B maintained for 4 min, 80% B to 100% B for 9 min and 100% B maintained for 14 min. The flow rate was 1 mL/min) in the positive ion MS mode; (**b**) CID spectrum of labeled 16:0/15:0 PC molecular species identified in the lipid mixture isolated from the outer membrane (*L. dumoffii* cultivated on the medium with labeled choline), recorded under NP LC/MS conditions (phase A—hexane/isopropanol (3:2, *v*/*v*) and phase B—an isopropanol/hexane/5 mM aqua solution of ammonium acetate (38:56:5, *v*/*v*/*v*). The following elution program was employed: from 53% B to 80% B for 23 min, 80% B maintained for 4 min, 80% B to 100% B for 9 min and 100% B maintained for 14 min. The flow rate was 1 mL/min) in the positive ion MS mode.

Figure 6 presents the fragmentation spectra of individual d_9_-labeled PC molecular species found in the inner and outer cell membranes recorded in the positive ion mode. In Figure 6a, the peaks at *m*/*z* 739.4, 615.4, and 593.4 corresponding to [M + Na − N(CD_3_)_3_]^+^, [M + Na − 192]^+^, and [M + Na − 214]^+^ confirmed the presence of the deuterated PC head group in the PC compound. Loss of the 20:0 and 15:0 fatty acids as RCOOH and RCOONa was observed in the CID spectrum. A similar fragmentation process was also observed in the CID spectra shown in Figure 6b, where the 16:0/15:0 PC molecular species was determined. In addition to the labeled PCs, unlabeled PCs *m*/*z* 706.9, 720.8, 734.9, 748.7, 762.9, 776.7, and 790.7 were identified in the inner and outer membranes.

To compare the amount of the unlabeled PC phospholipid class in *L. dumoffii* cell membranes (outer and inner), the Multiple Reaction Monitoring (MRM) mass spectrometry mode was employed. The MRM mode is defined as a sensitive and a selective mass spectrometry technique. In the first step of the technique, an ion of interest was selected and subjected to the fragmentation process. Then, only predefined and characteristic fragment ions were detected.

In our studies, the MRM pairs were prepared on the basis of fragmentation spectra of peaks at *m*/*z* 764.9 and 778.9 corresponding to the most intense PC signals in the negative ion mode in both cell membranes. The [M + CH_3_COO]^−^ ion and the characteristic fragment ions [M + CH_3_COO − 74]^−^ and [RCOO]^−^ of PCs obtained in the fragmentation process under the negative ion mode were chosen as MRM data (764.9/690.4, 764.9/241.2, and 778.9/704.4, 778.9/241.2). In this experiment, the MRM peak areas were compared and they showed higher values for the inner cell membranes (for the inner membrane: 764.9/690.4, peak area—1.02 × 10^6^, 764.9/241.2—1.47 × 10^6^; 778.9/704.4—2.08 × 10^6^, 778.9/241.1—3.18 × 10^6^; for the outer membrane: 764.9/690.4—4.85 × 10^5^, 764.9/241.2—6.47 × 10^5^, 778.9/704.4—8.57 × 10^5^, 778.9/241.1—1.31 × 10^6^). This indicated that the inner cell membrane seems to be richer in the PC phospholipid class.

### 2.3. Identification of the pcsA Gene in the L. dumoffii Genome

**Figure 7.**
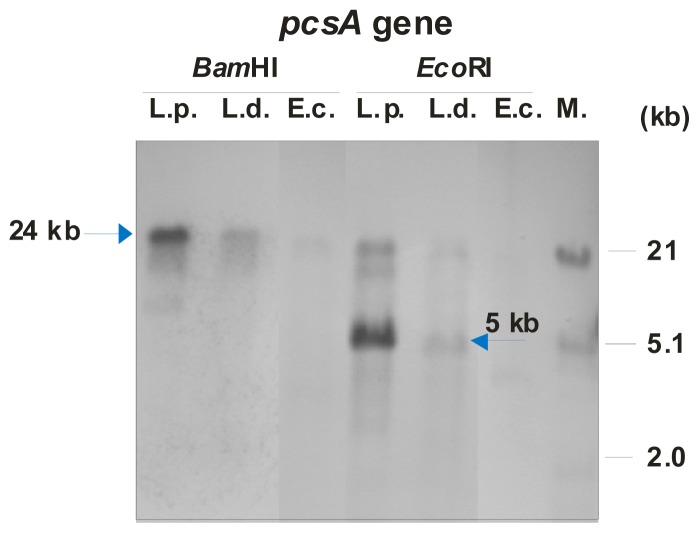
Identification of the *pcsA* gene in the *L. dumoffii* genome by Southern hybridization using a probe containing the *pcsA* gene of *L. pneumophila*. Hybridization was performed with genomic DNAs of *L. dumoffii* (L.d.), *L. pneumophila* (L.p.), and *E. coli* (E.c.) digested with *Bam*HI and *Eco*RI as described in Materials and Methods. M—molecular size standard (l DNA digested with *Hin*dIII and *Eco*RI).

Our biochemical analyses concerning PC synthesis in *L. dumoffii* indicated that this compound is produced in a one-step pathway, suggesting that a gene encoding phosphatidylcholine synthase is present in the genome of this bacterium. In order to identify the *pcsA* gene in *L. dumoffii*, Southern hybridization under low-stringency conditions was performed using genomic DNAs from *L. dumoffii*, *L. pneumophila*, and *E. coli* (as a negative control) digested with *Bam*HI and *Eco*RI. A DIG-labeled DNA fragment containing the *pcsA* of *L. pneumophila* was used as a probe (Figure 7). As a result, a homologue of *pcsA* was found in the *L. dumoffii* genome, although the intensity of detected signals was significantly lower in comparison to the signals obtained for *L. pneumophila*. The *pcsA* probe hybridised to a 24-kb-long *Bam*HI fragment and a 5-kb-long *Eco*RI fragment of *L. dumoffii.* In the case of *L. pneumophila*, strong positive signals were observed for the 24-kb *Bam*HI fragment and the 6-kb *Eco*RI fragment, respectively. On the contrary, only a slight unspecific signal was observed in hybridization with *E. coli* genomic DNA, confirming absence of a *pcsA* homolog in this genome (Figure 7).

In conclusion, these results showed that the *L. dumoffii* genome contained a *pcsA* gene; however, the low intensity of the observed signal suggested a low sequence identity between *L. pneumophila pcsA* and *L. dumoffii* homolog. This observation is in agreement with literature data that described high genetic and phenotypic diversity between *L. pneumophila* and other *Legionella* species [19].

To determine the nucleotide sequence of *L. dumoffii pcsA*, a set of degenerate primers was designed based on genomic sequences of a few *L. pneumophila* strains, *L. longbeachae* D-4968 and *L. drancourtii* LLAP12. Using these primers, a 900-bp-long PCR product of *L. dumoffii* was obtained and sequenced. In the assessment of the genetic relatedness between *L. dumoffii pcsA* and this gene in other sequenced *Legionella* species, the highest sequence identity to *L. bozemanae pcsA* (82%) and *L. longbeachae* (81%) was found. *L. dumoffii pcsA* showed lower sequence identity to *pcsA* of *L. drancourtii* (78%) and *L. pneumophila* ATCC 33155 (71%). The comparison of the *pcsA* sequence indicated a high level of diversity among the *Legionella* species, especially in 5′ and 3′ ends of *pcsA* genes. *L. dumoffii pcsA* encodes putative 254-aa-long phosphatidylcholine synthase, whose length was identical or very similar to the other *Legionella* PcsA proteins: *L. drancourtii* (254 aa), *L. longbeachae* (253 aa), and *L. pneumophila* (255 aa). In the upstream of the *L. dumoffii pcsA* coding sequence (−135 to −86 bp), a promoter sequence of high potential activity (*p* = 0.99) was identified (5′-actatttttatgttttttattttttgataaaacaacaatagtttagccca-3′). In addition, 6 nt upstream of the ATG translation start codon, a potential ribosome-binding site (AGGA) was found. The PcsA of *L. dumoffii* showed the highest sequence identity to the *L. bozemanae* homolog (90% identity and 96% similarity). Lower identity of this protein was established to PcsA of *L. longbeachae* (87% identity and 94% similarity), *L. drancourtii* (79% identity and 90% similarity), and *L. pneumophila* (74% identity and 84% similarity). The most conservative amino acid sequences were found in the central regions of PcsA proteins that are probably-functional domains of the enzyme, whereas their *N*- and *C*-termini are substantially more divergent.

### 2.4. The Cytotoxic Effect of L. dumoffii on THP-1 Cells

To determine the infectious doses of *L. dumoffii* for human macrophages (THP-1 cell line), the cytotoxic effect of different bacteria concentrations (expressed as the multiplicity of infection—MOI) was assessed with the MTT (3-(4,5 dimethyl-2-thiazolyl)-2,5-diphenyl-2H-tetrazolium bromide) method. The viability of the THP-1 differentiated cells incubated with *L. dumoffii* for 4 h at a MOI of 10, 50, 100, and 200 was found to be similar to that of the control cells (cell viability 103.05% ± 7.4%, 105.5% ± 8.0%, 101.6% ± 7.8%, 96.2% ± 7.2%, respectively). After 24-h incubation of THP-1 cells with the bacteria at a MOI of 100 and 200, a slight decrease in cell viability was observed (cell viability 99.2% ± 7.2%, 95.4% ± 7.4%, respectively). The analysis of the cytotoxic effect of *L. dumoffii* on THP-1 cells was also performed for the same doses of *L. dumoffii* cultured on choline-supplemented medium. No statistically significant differences of the toxic effect on the THP-1 cells were found between the bacteria cultured with and without choline. Based on these results and literature data [20,21], *L. dumoffii* at a MOI of 10 and 100 were chosen to study the TNF-α induction in the THP-1 differentiated cells.

### 2.5. TNF-α Induction by L. dumoffii in the THP-1 Differentiated Cells

#### 2.5.1. TNF-α Induction in the THP-1 Differentiated Cells by *L. dumoffii* Cultured on Choline-Supplemented and Non-Supplemented Medium

**Figure 8.**
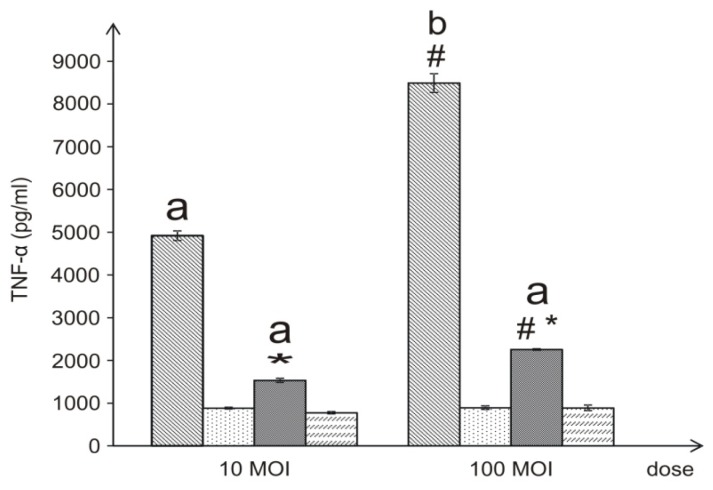
Effects of live and temperature-treated *L. dumoffii* cells cultured on the choline-supplemented medium and without choline on the ability to induce TNF-α production in THP-1 differentiated cells. Data represent the mean ± S.D. of five independent experiments. Control THP-1 differentiated cells (PMA-activated): TNF-α 22.6 ± 0.6 (pg/mL); Control non-activated THP-1 cells: TNF-α 18.32 ± 0.9 (pg/mL). # Significantly different from the lower bacteria dose, *p* ≤ 0.05; * Statistically significant in comparison to bacteria cultured on the choline non-supplemented medium, *p* ≤ 0.05 (ANOVA and Tukey’s *post hoc* test). ^a,b^ Statistically significant in comparison with similar experiments preformed with temperature-treated bacteria (^a^ *p* ≤ 0.05, ^b^ *p* ≤ 0.01) c (ANOVA and Tukey’s *post hoc* test) 
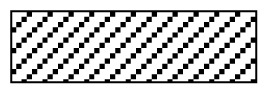
 —live *L. dumoffii;* 
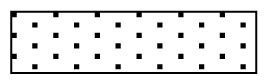
 —temperature-treated *L. dumoffii;* 
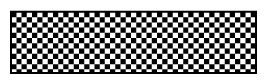
 —live *L. dumoffii* cultured on choline-supplemented medium; 
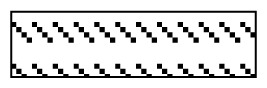
 —temperature-treated *L. dumoffii* cultured on choline-supplemented medium.

Levels of human TNF-α in the supernatants from the THP-1 experimental cultures were measured by an enzyme-linked immunosorbent assay (ELISA) after 4-h incubation of cells with live and temperature-killed *L. dumoffii* bacteria at the MOI of 10 or 100. To study the influence of choline on TNF-α production, both live and temperature-treated bacteria were obtained from BCYE medium supplemented with choline and without this compound. Regardless of the culture conditions, dose-dependent production of TNF-α was observed in all the experiments. However, statistically significant differences in obtained results were only noted when we considered the experiments with live bacteria. Bacteria cultured on the choline-supplemented medium, regardless of their concentration, induced TNF-α production at significantly lower level (*p* ≤ 0.05). However, inside study groups—live bacteria cultured with or without choline supplementation—level of TNF-α depended on bacteria concentration and was significantly higher when 100 MOI bacteria were used. The temperature-killed bacteria induced TNF-α at a considerably statistically significant lower level in comparison to live bacteria and presence of choline in the bacterial culture medium did not change the level of cytokine production (Figure 8).

#### 2.5.2. TNF-α Induction by the Outer and Inner Membrane of *L. dumoffii* in the THP-1 Differentiated Cells

**Figure 9.**
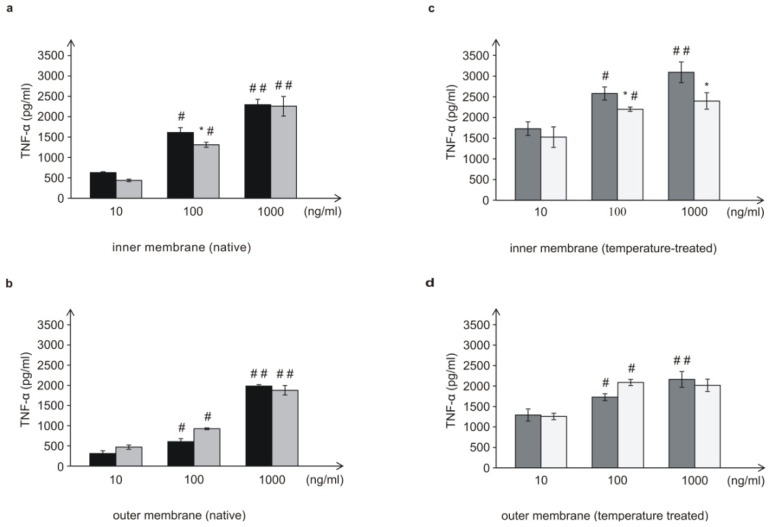
TNF-α (pg/mL) induction in the THP-1 differentiated cells by the outer and inner membrane of *L. dumoffii* (**a**,**b**,**c**,**d**). Control THP-1 differentiated cells (PMA-activated): TNF-α 22.6 ± 0.60 (pg/mL); Control non-activated THP-1 cells: TNF-α 18.32 ± 0.90 (pg/mL). Data represent the mean ± S.D. of five independent experiments; #—Significantly different from the lower (10 ng/mL) membrane concentration, *p* ≤ 0.05; ##—Significantly different from the lower (100 ng/mL) membrane concentration, *p* ≤ 0.05; *—Statistically significant in comparison to bacteria cultured on the choline non-supplemented medium, *p* ≤ 0.05 (ANOVA and Tukey’s *post hoc* test). 
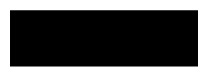
 —membrane from bacteria cultured on the choline non-supplemented medium. 
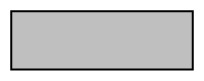
 —membrane from bacteria cultured on the choline-supplemented medium. 
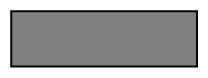
 —temperature-treated membrane from bacteria cultured on the choline non-supplemented medium. 
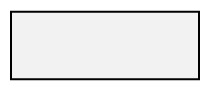
 —temperature-treated membrane from bacteria cultured on the choline-supplemented medium.

Inner and outer membranes (10, 100, and 1000 ng/mL concentrations) isolated from *L. dumoffii* cultured with and without choline were used for TNF-α induction (Figure 9). Both the inner and outer temperature-treated membranes induced TNF-α production in each dose and in a dose-dependent manner more efficiently than the temperature non-treated membranes. A comparison of the ability of the membranes to induce TNF-α showed that the inner membrane induced TNF-α more efficiently than the outer membrane, irrespective of the doses used and temperature treatment or no treatment.

Choline supplementation influenced TNF-α production in a different manner depending on the membrane type. The inner membrane isolated from choline-supplemented bacteria induced TNF-α less efficiently than the inner membrane of bacteria cultured without choline. However, only in the presence of 100 ng/mL of these membranes, the decrease in the TNF-α production was statistically significant (Figure 9A,C). In turn, the same concentration of the outer membranes of choline-supplemented bacteria (both, non- and temperature-treated) caused opposite effect—slightly increased the level of TNF-α in comparison with the outer membranes from bacteria cultured without choline (Figure 9B,D).

### 2.6. In Vitro Infection of Differentiated THP-1 Cells with L. dumoffii

To compare the levels of *L. dumoffii* internalization by differentiated THP1 cells, bacteria cultured on the medium with and without choline were incubated with macrophages for 2 h. Next, bacteria that were not phagocytised by macrophages were killed by gentamycin treatment. Only intracellular bacteria released after macrophage lysis were seeded onto the BCYE agar medium. The number of colonies was higher for bacteria cultured with choline before the internalization process (171.8 ± 4.2 for the choline-grown bacteria, 143.2 ± 3.8 for the non-choline grown bacteria). This investigation indicates that choline-grown bacteria undergo association by macrophages more readily than non-choline grown bacteria. However, the results were not statistically significant. *L. dumoffii* did not replicate in differentiated THP1 cells irrespective of the bacterial culture conditions (data not shown).

## 3. Discussion

Our previous and current investigations showed that *L. lytica*, *L. bozemanae*, and *L. dumoffii* form PC [11,22]. In this study, the ability of *L. dumoffii* to synthesize PC in a choline-dependent manner was investigated. It was evidenced that the bacteria used exogenous choline for PC synthesis via the Pcs pathway. The main PC d9-PC[31:0 + H]^+^, identified on the MALDI/TOF spectrum and synthesised via this pathway, was confirmed by LC/ESI-MS as PC 16:0/15:0, also identified in *L. bozemanae*. Both species exhibit blue-white fluorescence; and the presence of PC 16:0/15:0 is an additional chemotaxonomic feature that classifies both species as representatives of the genus *Fluoribacter* in the *Legionellaceae* family [23]. The Pcs pathway in *L. dumoffii* was confirmed by identification of the *pcsA* gene encoding the phosphatidylcholine synthase. All *Legionella* spp. genomes characterized so far contain the *pcsA* gene encoding phosphatidylcholine synthase, suggesting a significant function of this protein in their metabolism. Previously, we have identified the *pcsA* gene in *L. bozemanae* and found that PC is effectively produced in a one-step pathway, in which the PcsA enzyme is engaged [11]. Likewise, the *L. dumoffii* PcsA presented in this study and *L. bozemanae* PcsA exhibited the highest sequence amino acids identity to the *L. longbeachae* PcsA, which confirmed genetic relatedness of these three *Legionella* species.

The presence of unlabeled PC in the MALDI-TOF spectrum obtained from lipids of bacteria cultured on choline has indicated that *L. dumoffii* forms PC also by triple PE methylation. LC/ESI-MS analysis of PC species present in both membranes labeled with a deuterated precursor allowed us to distinguish PC species synthesized from the CDP-choline pathway and the PE methylation pathway. Our previous investigations showed that *L. bozemanae* produced PC via two independent pathways PmtA and Pcs [11]. Similarly, both PC synthesis pathways function in *L. pneumophila*. The pathway in which *Legionella* spp. utilize exogenous choline is dominant and seems to be more energetically efficient [10]. However, in the genetic experiments that were conducted (hybridization, PCR analyses using several degenerative primers and amplicon sequencing), we were unable to identify a *pmtA* homologue in the *L. dumoffii* genome (data not shown). Both PC synthesis pathways have also been reported in legume endosymbionts (*Rhizobium leguminosarum*, *Sinorhizobium meliloti*, *Bradyrhizobium japonicum*) and in the plant pathogen *Agrobacterium tumefaciens*. In human pathogens such as *Pseudomonas aeruginosa*, *Brucella melitensis*, and *Borrelia burgdorferi*, PC is produced only via the Pcs pathway [24].

Several studies have shown that the PC of bacterial membranes can be important to host-associated bacteria in pathogenesis and symbiosis. Our results have indicated that bacteria cultured on choline undergo internalization by THP1 macrophages more readily than bacteria cultured on non-choline-supplemented medium. The increase in internalization in the case of bacteria cultured on choline was not statistically significant, which may suggest a low level of participation of PC species in this process, although other papers indicate that some bacterial PC derivatives play a direct role in association with macrophages [25].

PC-deficient mutants of *B. japonicum* and *S. meliloti* were characterized by substantially reduced symbiosis with their plant hosts [26,27]. PC is indispensable for the plant pathogen *A. tumefaciens* to assembly T4SS components, important factors in formation of plant crown-gall tumors [28,29]. A *Brucella abortus pcs* mutant exhibited an altered cell envelope; therefore, it did not establish a replication niche inside the macrophages. Additionally, it showed a severe virulence defect in a murine model of infection [30,31].

A PC-deficient *P. aeruginosa* mutant exhibited the same level of sensitivity to antibiotics and antimicrobial peptides as wild strains. PC deficiency did not change the mobility and capability of biofilm formation on an abiotic surface. However, PC may have a specific role in the interaction with eukaryotic hosts, e.g., it might aid in assembly or localization of specific proteins in *P. aeruginosa* [32].

*L. dumoffii* cultured on choline-supplemented medium exhibited altered sensitivity to *Galleria mellonella* antimicrobial defense factors such as defensin and apoLp-III [33]. Replacement of PE with PC induced concurrent structural and functional changes in the ABC multidrug exporter of *Lactococcus brevis* [34].

The membrane fraction experiment and LC/ESI-MS analyses suggest that PC in *L. dumoffii* is present in the outer and inner membranes. In *L. pneumophila* and *P. aeruginosa*, PC was also localized in both membrane compartments [35,36]. MRM analysis showed that the amount of PC in the inner membrane of *L. dumoffii* was higher than in the outer membrane. Moreover, there were differences in the structure of the PC species present in bacteria cultured with and without choline. These differences might be important for interactions of bacteria with host cells.

Several lines of evidence indicate that PC is able to modulate the inflammatory functions of monocytic cells. Tonks *et al.* showed that PC 16:0/16:0 significantly inhibited TNF-α release from the human monocytic cell line MonoMac-6 in a dose-dependent manner. In contrast, PC 20:4/16:0 did not reduce TNF-α, which indicated that regulation of the inflammatory response was associated with the composition of fatty acids forming PC [37]. In our study, live *L. dumoffii* bacteria cultured on the choline-supplemented medium, induced three times less TNF-α than the cells grown on the non-supplemented medium. Similarly, the inner (temperature-treated and -untreated) membranes isolated from bacteria cultured on choline, applied at almost all the doses, induced a decreased level of TNF-α, in comparison to bacteria cultured without choline. Bacteria grown on choline incorporate into their inner membranes PC species with longer fatty acids than bacteria cultured without choline, which may have a significant effect on the lower induction of TNF-α level. In comparison to live bacteria, temperature-treated bacteria induced significantly lower TNF-α level. It is connected with the fact, that in live Legionella HSP, flagellin, and LPS are the main inducer of TNF-α. Temperature heating (90 °C, 20 min) causes HSP and flagellin degradation, therefore still present LPS is only inducer of this cytokine. This may be similar to the case of *L. pneumophila* LPS [38], although it is not known what the efficiency of *L. dumoffii* LPS in the induction of TNF-α is, since no such investigations have been carried out and the structure of *L. dumoffii* LPS is not known.

In the case of the 10- and 100-ng/mL concentrations of the outer membrane of live bacteria cultured on choline, TNF-α induction was higher than in the case of bacteria without choline; however, these differences were not statistically significant. It has been shown that lipopolysaccharide (LPS) of Gram-negative bacteria, a well-known TNF-α inducer located in the outer membrane, had an influence on the level of the cytokine as well. However, *L. pneumophila* LPS is about 1000 times less potent in its ability to induce pro-inflammatory cytokines (TNF-α, IL-1β, IL-6, IL-8) in Mono Mac 6 cells than the LPS of the *Enterobacteriaceae* members [21]. Cao *et al*. showed that addition of PC18:2/18:2 into the culture of Kupffer cells significantly reduced LPS-stimulated TNF-α generation [39]. We did not observe such an effect. Probably, the PC structure has a significant impact on the level of induction of this cytokine.

## 4. Experimental Section

### 4.1. Bacterial Strain and Growth Conditions

*L. dumoffii* strain ATCC 33279, *L. pneumophila serotype 3* ATCC 33155 were cultured on buffered charcoal-yeast extract (BCYE) agar plates, which contained a *Legionella* CYE agar base (Oxoid, Basingstoke, Hampshire, UK) supplemented with the Growth Supplement SR0110A (ACES buffer/potassium hydroxide, ferric pyrophosphate, l-cysteine HCl, α-ketoglutarate; Oxoid, Basingstoke, Hampshire, UK) for three days at 37 °C in a humid atmosphere and 5% CO_2_ [40]. *L. dumoffii* were also cultivated on this medium enriched with 100 μg·mL^−1^ of choline-trimethyl-d_9_ chloride (Sigma-Aldrich, Steinheim, Germany). The bacteria collected from this medium were washed three times with water by intensive vortexing and centrifugation at 8000× *g* for 10 min. Bacteria cultured with and without choline were killed by 90 °C for 20 min. The efficiency of temperature inactivation of bacteria was checked by streaking the bacteria on BCYE plates. *Escherichia coli* DH5α strain was grown in Luria-Bertani (LB) medium at 37 °C [41].

### 4.2. Fractionation of L. dumoffii Cultured on BCYE Medium with and without Choline

The bacteria were collected from 12 BCYE plates supplemented with labeled choline and 12 non-supplemented BCYE plates. They were washed twice in saline in order to remove the remaining medium. Cell membrane isolation was performed essentially as described by Hindahl and Iglewski [35].

The cells were washed twice with cold 10 mM HEPES (*N-*2-hydroxyethylpiperazine- *N*-2-ethanesulfonic acid; Sigma-Aldrich, Steinheim, Germany) buffer (pH 7.4) and centrifuged at 8000× *g*, 15 min in 4 °C. The cell pellets were suspended in 15 mL of 10 mM HEPES buffer containing 20% sucrose (*w*/*v*) and incubated with DNase (0.3 mg) (Sigma-Aldrich, Steinheim, Germany) and RNase (0.3 mg) (Sigma-Aldrich, Steinheim, Germany) at 37 °C for 30 min 0.8-mL suspensions were lysed by three passages through a French press (SLM-Amico Instruments, Thermo Spectronic, Rochester, NY, USA) at 18000 lb/in^2^. After centrifugation for 20 min at 1000× *g*, 4 °C performed to remove cell debris and undisrupted cells, total membrane fractions were collected by centrifugation for 60 min at 100,000× *g*, 4 °C (SW 32Ti rotor, Beckman Coulter, Brea, CA, USA). Next, they were washed twice in cold 10 mM HEPES buffer by centrifugation at 100,000× *g* for 1 h, 4 °C, the pelleted membranes were suspended in 2.5 mL of 10 mM HEPES buffer and layered onto a seven-step sucrose gradient.

### 4.3. Isolation of the Outer and Inner Membranes

Two milliliters of each cell membrane suspension were loaded on the top of a discontinuous gradient prepared by combining sucrose solutions of the following concentrations: 6 mL 70%, 9 mL 64%, 8 mL 58%, 5 mL 52%, 4 mL 48%, 3mL 42%, 3 mL 36% (*w*/*v*) in 10 mM HEPES buffer, pH 7.5. The gradient was centrifuged at 114,000× *g*, 20 h, 4 °C (SW 32Ti rotor, Beckman Coulter, Brea, CA, USA), and 1 mL fractions were subsequently collected from the top of the centrifugation tube. The protein content was determined in the individual fractions and the fractions from the upper band of the gradient and the lower band of the gradient were collected separately. Next, the upper and lower fractions pooled separately were suspended in 10 mM HEPES buffer and centrifuged at 54,000× *g* for 1 h (MLA 80, Optima MAX-XP, Beckman Coulter, Brea, CA, USA). The membrane pellets were washed three times in cold deionized water and centrifuged at 54,000× *g* for 1 h (MLA 80, Optima MAX-XP, Beckman Coulter, Brea, CA, USA). Next, they were diluted in 250 μL of water (MQ, Millipore, Billerica, MA, USA) for further analyses, *i.e*., enzyme assays and lipid isolation.

The efficacy of the membrane isolation procedure was confirmed by measuring the activity of NADH oxidase [42] and esterase. The concentration of protein in the fractions was determined using the Bradford method and bovine serum albumin as a standard [43].

### 4.4. Enzyme Assays

As marker enzymes for the inner and outer membrane, activities of NADH oxidase and esterase, respectively, were determined by spectrophotometric analysis of the absorbance decrease at 340 nm for NADH oxidase and the absorbance increase at 405 nm for esterase. For NADH oxidase, 62 μL of the reaction mixture (50 mM Tris/HCl, pH 7.5, 0.2 mM DTT, 0.12 mM NADH) (Sigma-Aldrich, Steinheim, Germany) was incubated for 15 min at 37 °C with 8 μL of each fraction in a microtitre plate. For esterase activity, 63 mg of *p*-nitrophenyl acetate (Sigma-Aldrich, Steinheim, Germany) was dissolved in 10 mL of ethanol. One milliliter of this solution was slowly added to 100 mL of distilled water. Ten microliters of each fraction were added to 90 μL of the substrate solution, incubated for 10 min at 25 °C, and absorbance at 405 nm was recorded. Fractions with the NADH oxidase and esterase activities representing the inner and outer membrane fractions were lyophilized and weighed.

### 4.5. Isolation of Lipids

Lipids were isolated from bacterial cells cultivated on BCYE medium enriched with labeled choline using the Bligh and Dyer (1959) method: chloroform/methanol (1:2 *v*/*v*) [44]. Lipids were analyzed with MALDI/TOF mass spectrometry.

Lipids from the inner and outer membranes prepared from bacteria grown on the choline-supplemented and non-supplemented medium were extracted using the same extraction method. The dried organic phase was then purified with a mixture of hexane/isopropanol (3:2, *v*/*v*). The extracts were dried under nitrogen before weighing and then dissolved in chloroform for further LC/ESI-MS analysis.

### 4.6. Matrix-Assisted Laser-Desorption/Ionization (MALDI)-Time of Flight (TOF) Mass Spectrometry

MALDI-TOF mass spectrometry analysis was performed on a Voyager-Elite instrument (PE Biosystems, Foster City, CA, USA) using delayed extraction in the reflectron mode. The dry lipid extract was dissolved in a CHCl_3_/CH_3_OH mixture (2:1, *v*/*v*). The sample constituents mixed with 0.5 M aniline salt of α-cyano-4-hydroxycinnamic acid (CHCA) as a matrix were desorbed and ionized with a nitrogen laser at an extraction voltage of 20 kV. Angiotensin was used as an internal standard. Each spectrum was the average of about 256 laser shots.

### 4.7. Liquid Chromatography Coupled with the Mass Spectrometry Technique Using the Electrospray Ionization Technique (LC/ESI-MS)

The LC/ESI-MS analyses were performed on a Prominence *LC-20* (Shimadzu, Kyoto, Japan) liquid chromatograph coupled with a tandem mass spectrometer 4000 Q TRAP (Applied Biosystems Inc., Foster City, CA, USA), equipped with an electrospray (ESI) ion source (TurboIonSpray, Applied Biosystems Inc., Foster City, CA, USA) and a triple quadrupole/linear ion trap mass analyzer. The separation of the phospholipid mixture was carried out by normal phase chromatography using a 4.6 × 150 mm Zorbax SIL RX column (Agilent Technologies, Palo Alto, CA, USA). Hexane/isopropanol (3:2, *v*/*v*) was used as solvent A and an isopropanol/hexane/5 mM aqua solution of ammonium acetate (38:56:5, *v*/*v*/*v*) as solvent B. The following elution program was employed: from 53% B to 80% B for 23 min, 80% B maintained for 4 min, 80% B to 100% B for 9 min and 100% B maintained for 14 min. The flow rate was 1 mL/min. The lipid extracts were dissolved in phase A.

To identify PC species, the precursor ion mode (PI) and neutral loss scan (NL) mass spectrometry techniques were employed in the positive ion mode. The measurements were performed using an electrospray ion source (ESI) with the following parameters: ion spray voltage (IS)—5500 V, declustering potential (DP)—40 V, and entrance potential (EP)—10 V. Nitrogen was used as a curtain (CUR 20 psi), a nebulizer (GS1 50 psi), and a collision gas. The source temperature was set at 250 °C. The collision-induced dissociation (CID) spectra were obtained in the positive and the negative ion mode using collision energy of 50 eV.

The Multiple Reaction Monitoring (MRM) technique performed in the negative ion mode was employed to compare the PC amounts in the analyzed cell membranes. The most abundant PC molecular species at *m*/*z* 778.9 (16:0/15:0) and 764.9 (15:0/15:0) were chosen in this experiment. Based on the fragmentation spectra of the selected peaks in the negative ion mode, the following MRM pairs were used: 764.9/690.4, 764.9/241.2, and 778.9/704.4, 778.9/241.2.

Chemicals for the LC/MS system (hexane, isopropanol, acetonitrile LC grade) were obtained from Merck (Darmstadt, Germany), and ammonium acetate from Sigma-Aldrich Fluka (Steinheim, Germany).

### 4.8. DNA Methods, PCR Amplification, and Southern Hybridization

Restriction enzyme digestion, agarose gel electrophoresis, DNA labeling, and Southern hybridization were used for genomic DNA isolation [41]. To amplify the DNA fragment containing *pcsA* of *L. pneumophila* serotype 3 (ATCC 33155), the PCR reaction was performed using 100 ng of genomic DNA, REDTaq ReadyMix (Sigma-Aldrich, Steinheim, Germany) and 0.2 μM of each forward (pcsF: 5′-CTCTAGGATCCGTAATGAATCCAATAAA-3′) and reverse (pcsR: 5′-CATAAATTGG ATCCAAACTCAATCTTTATTAT-3′) primers in a 50-μL final volume. The PCR reaction was performed with the following temperature profile: initial denaturation at 94 °C for 4 min, 30 cycles of denaturation 94 °C for 1 min, annealing at 48 °C for 40 s, extension at 72 °C for 60 s, and final extension at 72 °C for 4 min. This PCR fragment was labeled using the non-radioactive DIG DNA Labeling and Detection kit according to the manufacturer’s instruction (Roche Applied Science, Penzberg, Germany). The 800-bp-long DIG-labeled amplicon with the *pcsA* gene was used as a probe in the Southern hybridization. Additionally, DIG-labeled DNA of phage λ digested with *Eco*RI and *Hin*dIII restriction enzymes were used as a molecular size marker. Genomic DNA from *L. pneumophila* serotype 3, *L. dumoffii*, and *E. coli* DH5α (used as a negative control) were digested with *Bam*HI and *Eco*RI enzymes, separated by 0.7% agarose gel electrophoresis, and blotted. In addition, λ DNA digested with both *Eco*RI and *Hin*dIII enzymes was used as molecular markers. The hybridization experiments were performed at reduced-stringency conditions at 37 °C using 20% formamide in pre-hybridization and hybridization solutions as described previously [11].

To amplify and sequence the *L. dumoffii pcsA* gene, a set of degenerate primers has been designed based on the genomic sequences of *L. pneumophila* strain Philadelphia 1, *L. longbeachae* D-4968, and *L. drancourtii* LLAP12. Primers pcsLd-F (5′-ACTTTTKATWATYGATRMTATTTT-3′) and pcsLd-R (5′-TAATCATWAAADABYCAAAGTCTAT-3′) allowed amplification of the longest 900-bp fragment containing *pcsA* gene. The PCR reactions were performed with the temperature profile described above, except the annealing temperature that was decreased to 43 °C. The amplified DNA fragment was purified on the columns (A&A Biotechnology, Gdynia, Poland) and sequenced using the BigDye terminator cycle sequencing kit (Applied Biosystems, Inc., Foster City, CA, USA) and the ABI Prism 310 sequencer. Database searches were done with the BLAST and FASTA programs available at the National Center for Biotechnology Information (Bethesda, MD, USA) and the European Bioinformatics Institute (Hinxton, UK). The sequence of *L. dumoffii pcsA* obtained in this study has been deposited in NCBI GenBank under accession number KC197708. Promoter prediction in the upstream region of *L. dumoffii pcsA* was done using BDGP Neural Network Promoter Prediction (fruitfly.org).

### 4.9. THP-1 Cell Culture

The human acute monocytic leukemia *cell line* (THP-1) was obtained from the European Collection of Cell Cultures (Cat No. 88081201). Cells were cultured at (0.5–7) × 10^5^ cells/mL in RPMI 1640 supplemented with 10% heat-inactivated fetal calf serum (FCS), 10 mM HEPES, 2 mM glutamine, 100 U/mL penicillin, and 100 μg/mL streptomycin. The cells cultured in tissue culture flasks (Falcon, Bedford, MA, USA) were incubated at 37 °C in a humidified atmosphere of 5% CO_2_. The culture media, antibiotics, and FCS were purchased from Sigma-Aldrich (Steinheim, Germany).

### 4.10. THP-1 Cell Differentiation

THP-1 were seeded onto 24-well plastic plates (Nunc, Roskilde, Denmark) at a density of 5 × 10^5^ cells/well in RPMI 1640 supplemented with 10% FCS, and treated with a final concentration of 50 ng/mL phorbol 12-myristate 13-acetate (PMA) (Sigma-Aldrich, Steinheim, Germany) for three days to induce maturation toward adherent macrophage-like cells. Subsequently, unattached cells were removed and after three-time washing adherent THP-1 cells were cultured in medium without PMA for three consecutive days with daily fresh medium change.

### 4.11. THP-1 Cell Viability Assay

The viability of THP-1 cells exposed to *L. dumoffii* bacteria was determined by the MTT assay, in which the yellow tetrazolium salt 3-(4,5 dimethyl-2-thiazolyl)-2,5-diphenyl-2H-tetrazolium bromide (MTT) is metabolized by viable cells to purple formazan crystals. The cells at a density of 5 × 10^4^ cells/well were seeded onto 96-well plates (Nunc, Roskilde, Denmark) and cultured in 10% RPMI 1640 for 24 h. Next, the medium was replaced with a fresh one with addition of 2% FCS and different concentrations of *L. dumoffii* at a MOI of 10, 50, 100, and 200. The treated cells were maintained in a humidified CO_2_-incubator at 37 °C for 4 and 24 h. Subsequently, MTT solution (25 μL of 5 mg/mL in PBS) (Sigma-Aldrich, Steinheim, Germany) was added to each well. The cells were incubated at 37 °C for 3 h, and 100 μL of SDS in 0.01 M HCl was added to dissolve the formazan crystals during overnight incubation. The controls included native (non-treated) cells and the medium alone. The spectrophotometric absorbance was measured at 570 nm wavelength using a VICTOR X4 Multilabel Plate Reader (Perkin Elmer, Waltham, MA, USA). The data are presented as percentage of control cell viability.

### 4.12. In Vitro Infection of Differentiated THP-1 with L. dumoffii—Control of Internalization and Intracellular Growth

Differentiated THP-1 cells (as described in 4.10.) were infected with 10 MOI of live *L. dumoffii* cultured on choline-supplemented and non-supplemented medium. After incubation for 2 h at 37 °C, 5% CO_2_, nonphagocytized bacteria were killed by the addition of 100 mg of gentamicin/mL for 1 h. Next, supernatants were removed and the macrophages were washed three-times with PBS. 1 mL of sterile distilled water (for bacterial internalization study) or 1 mL of RPMI 1640 supplemented with 10% FCS (without antibiotics) was added and the macrophages were incubated for 24, 48, and 72 h (intracellular bacterial growth study).

#### 4.12.1. Bacteria Internalization Assay

Cells suspended in 1 mL of sterile distilled water were disrupted by aspiration through a 25-gauge needle and then series of 10-fold dilutions were made. Subsequently, 0.1 mL of each dilution was inoculated onto BCYE agar and colonies of culturable *L. dumoffii* were counted after three days of incubation at 37 °C, 5% CO_2_.

#### 4.12.2. Intracellular Bacteria Growth Assay

After 24, 48, and 72 h of cell culture incubation, supernatants were collected into sterile tubes, centrifuged (8000 rpm/min for 10 min), and washed with sterile distilled water. One milliliter of sterile distilled water was added to THP-1 cells, which were disrupted as described above and pooled with centrifuged pellet of the respective supernatant. 0.1 mL of series of 10-fold dilutions of each sample were inoculated onto BCYE agar and incubated as above.

Formation of colonies was determined in triplicate for at least two independent experiments.

### 4.13. Induction of TNF-α with L. dumoffii in THP-1 Cells

The differentiated macrophages were treated with live or dead *L. dumoffii* for 4 h at a MOI = 10 or 100. In another experiment, the macrophages were treated with different concentrations (10–1000 ng/mL) of *L. dumoffii* outer or inner membranes non-treated and treated with temperature (90 °C, 20 min).

Additional controls were performed: non-treated macrophages, non-activated THP-1 cells.

In all experiments, the bacteria had been previously cultured on medium with or without addition of choline (choline-trimethyl-d_9_ chloride, Sigma-Aldrich, Steinheim, Germany). After incubation for 4 h at 37 °C, 5% CO_2_, cell culture supernatants were collected and frozen immediately at −80 °C for further TNF-α determination. TNF-α level was measured by the ELISA method using a commercial kit from R&D Systems according to the manufacturer’s instructions (R&D Systems Inc., Minneapolis, MN, USA). All experiments were conducted in five independent replicates.

### 4.14. Statistics

Values are expressed as mean ± S.D. Results were statistically evaluated using two-way ANOVA and Tukey’s *post hoc* tests (Statistica software ver. 6.0, StatSoft Inc., Tulsa, OK, USA, 2001). *p* values of ≤0.05 were considered significant.

## 5. Conclusions

We have demonstrated that *L. dumoffii* has an ability to utilize extracellular choline in the Pcs pathway, which may be a dominant pathway, as indicated by the presence of a strong promoter for the phosphatidylcholine synthase gene. The use of the LC/ESI-MS technique allowed us to provide evidence that the PCs synthesized by *L. dumoffii* are localized in both the inner and outer membranes, but they are structurally different. This technique allowed identification of the PC species in total lipid extracts isolated from live bacteria without any chemical modification, thereby showing the physiological state of the cells. The structural differences in the PC species affect the induction of TNF-α—a key player in immuno-inflammatory response. In the presence of choline, the bacteria exhibited a reduced capability of TNF-α induction. The reduction of inflammatory response caused by PC species might collaborate to evade the immune system, thus, allowing *L. dumoffii* to establish in the host. The identification of selective inhibitors of phosphatidylcholine synthase may contribute to development of specific adjuvant therapy in treatment of legionellosis.

## Figures and Tables

**Table 1.** The major d_9_-labeled phosphatidycholine (PC) species determined by Liquid Chromatography Coupled with the Mass Spectrometry Technique Using the Electrospray Ionization Technique (LC/ESI-MS) in the inner and outer membrane of *L. dumoffii* cells.

*m*/*z*	PC structure, Sn-1/Sn-2 a	

	Inner membrane	Outer membrane
737.8	d_9_-PC15:0/15:0	d_9_-PC15:0/15:0
	d_9_-PC14:0/16:0	
751.9	-	d_9_-PC16:0/15:0
777.8	d_9_-PC16:0/17:1	-
791.9	d_9_-PC17:0/17:1	d_9_-PC17:0/17:1
807.9	d_9_-PC20:0/15:0	-
819.9	d_9_-PC20:0/16:1	-

a 17:1 or cyclic 17:0, indistinguishable under the present mass spectrometry conditions.
